# Use of Direct Oral Anticoagulants in Patients With Upper Extremity Deep Vein Thrombosis: A Meta-Analysis of Efficacy and Safety

**DOI:** 10.7759/cureus.83570

**Published:** 2025-05-06

**Authors:** Ayman Zyada, Ayman Fakhry, Sohiel Nagib, Omar Alnadi, Ahmed Abouelseoud, Rahma Seken, Muhammad Jabr

**Affiliations:** 1 Vascular Surgery, University Hospitals of Leicester NHS Trust, Leicester, GBR; 2 Vascular Surgery, Egyptian Military Medical Academy, Alexandria, EGY; 3 Vascular Surgery, Royal Vascular Center, Alexandria, EGY; 4 General Surgery, Abo Qir General Hospital, Alexandria, EGY; 5 Vascular Surgery, Alexandria Main University Hospital, Alexandria, EGY; 6 Faculty of Medicine, Al-Azhar University, Damietta, EGY; 7 Medicine and Surgery, Abu Qir General Hospital, Alexandria, EGY

**Keywords:** anticoagulation therapy, bleeding risk, direct oral anticoagulants, doacs, thrombosis management, upper limb deep vein thrombosis, venous thromboembolism

## Abstract

Upper extremity deep vein thrombosis (UEDVT), distinct in etiology from lower limb DVT, often arises from catheter use, malignancy, or thoracic outlet syndrome. While direct oral anticoagulants (DOACs) are established for lower limb DVT, their role in UEDVT remains understudied. This meta-analysis evaluates the efficacy and safety of DOACs compared to low-molecular-weight heparin (LMWH) in UEDVT. A systematic PubMed search identified nine studies (643 DOAC-treated patients). Outcomes included mortality, venous thromboembolism (VTE) recurrence, pulmonary embolism (PE), and major bleeding.

DOACs demonstrated significantly lower mortality (2.49% vs. 16.5-27.5%; p<0.001), VTE recurrence (0.93% vs. 5%; p<0.001), and PE incidence (0.31% vs. 5-8%; p<0.001) compared to historical LMWH data. However, major bleeding rates were higher with DOACs (2.02% vs. 0.25%; p<0.001). Patient cohorts predominantly had cancer-related (66.7%) or catheter-associated (64.5%) UEDVT, with rivaroxaban being the most used DOAC (70.9%). Median treatment duration was three months, with a six-month follow-up.

These findings suggest DOACs may offer superior efficacy in reducing mortality and thrombotic complications in UEDVT, though with an increased bleeding risk. Limitations include reliance on historical LMWH comparisons, heterogeneity in study designs, and small event counts for PE. Standardized imaging and extended follow-up are needed to assess long-term outcomes. While DOACs present a promising alternative, cautious use in high-bleeding-risk patients is warranted. Further randomized trials are essential to validate these results and refine clinical guidelines.

## Introduction and background

Direct oral anticoagulants (DOACs, a class of oral blood-thinning medications) are approved for the treatment of lower limb deep vein thrombosis (DVT) and have been extensively studied in comparison to traditional anticoagulants in terms of efficacy and safety [1,2]. However, evidence regarding their use in upper extremity DVT (UEDVT, blood clots in the deep arm/shoulder veins) remains scarce.

UEDVT can lead to life-threatening complications such as pulmonary embolism (PE) if untreated, and it presents distinct characteristics compared to lower limb DVT. While some cases share common risk factors, such as malignancy, hereditary and acquired thrombophilia, sepsis, pregnancy, and immobility associated with fractures [3,4], UEDVT is frequently secondary to conditions such as central venous catheter placement, iatrogenic injuries, axillary lymphadenopathy, or thoracic outlet syndrome (TOS).

DOACs may behave differently in UEDVT compared to lower limb DVT due to distinct pathophysiological and clinical factors. UEDVT is often catheter-related or cancer-associated, involving fibrin-rich thrombi and persistent prothrombotic stimuli, which may respond less effectively to DOACs. Additionally, faster venous flow, altered hemodynamics, and the presence of foreign bodies like catheters can impact drug distribution and clot resolution. In cancer patients, drug interactions and altered pharmacokinetics further complicate DOAC use. Unlike lower limb DVT, UEDVT lacks strong clinical trial data supporting DOAC efficacy, making low-molecular-weight heparin (LMWH) the preferred treatment in many such cases.

Current guidelines provide recommendations for the management of UEDVT. The European Society for Vascular Surgery (ESVS) guidelines suggest anticoagulation therapy unless major contraindications are present, with LMWH, an injectable anticoagulant, followed by vitamin K antagonists as the conventional regimen [5]. Similarly, the American College of Chest Physicians (ACCP) guidelines recommend anticoagulation for a minimum duration of three months [6].

The primary aim of this study is to assess the efficacy of DOACs in the treatment of UEDVT and to compare their outcomes with those reported for traditional anticoagulant therapies. This study was previously presented at the International Vein Congress (IVC Miami 2025) on April 26, 2025.

Method

A meta-analysis evaluated existing literature on UEDVT cases treated with DOACs, comparing their clinical outcomes with those of conventional anticoagulant therapies. A systematic literature search was performed in MEDLINE using MESH keywords: "DOAC", "AND", "upper limb DVT", following the PRISMA (Preferred Reporting Items for Systematic Reviews and Meta-Analyses) guidelines, without imposing language restrictions up to February 21, 2025. Manual screening of reference lists from retrieved articles was conducted to identify additional eligible studies. Inclusion criteria comprised confirmed diagnoses of UEDVT, treatment with DOACs, and documented follow-up completion. Exclusion criteria encompassed studies involving thrombolytic therapy, incomplete follow-up data, no reported clinical outcomes in anticoagulated patients, and case reports or case series with fewer than 10 participants.

**Figure 1:**
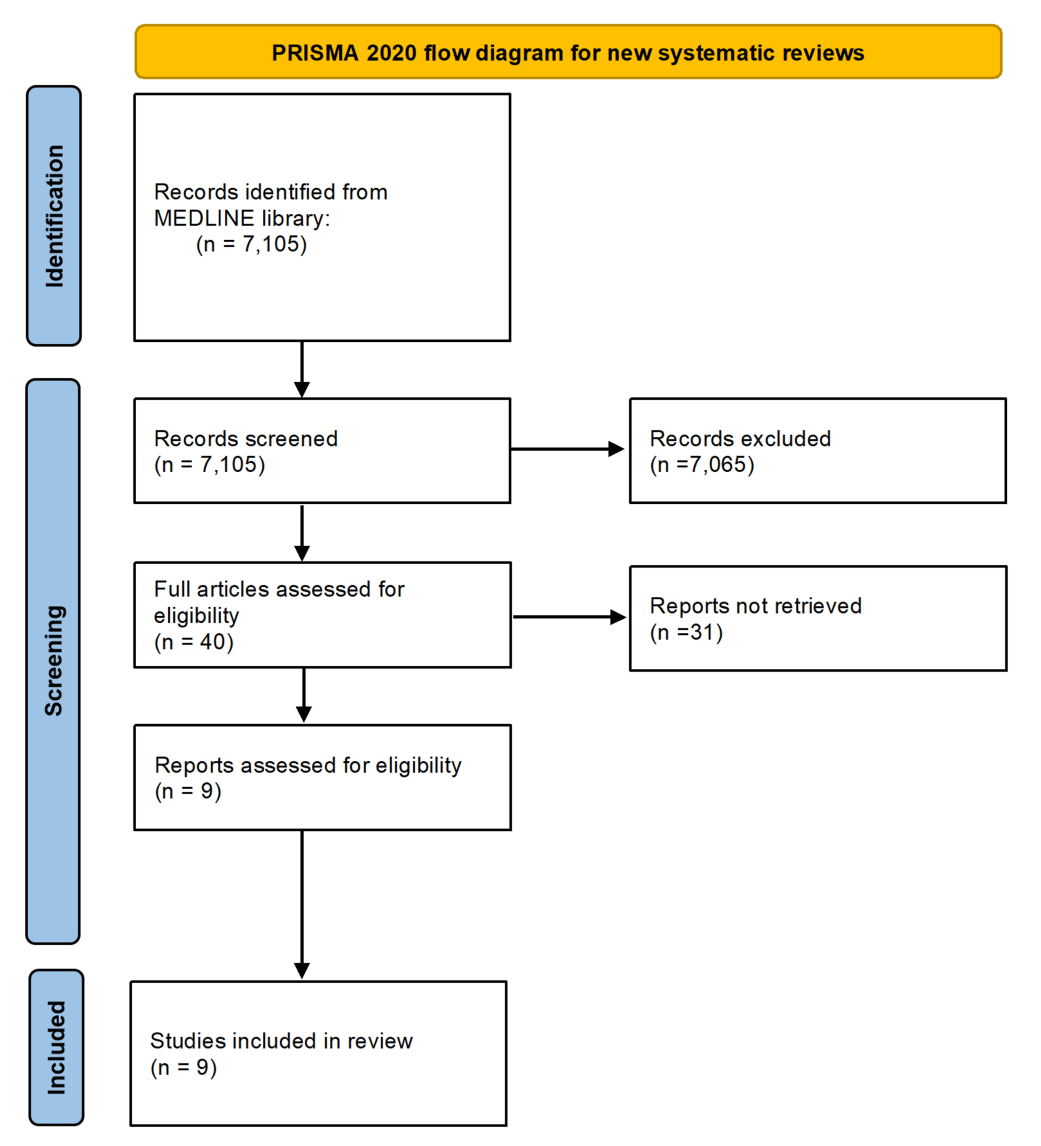
PRISMA flow chart PRISMA, Preferred Reporting Items for Systematic Reviews and Meta-Analyses

From the initial 7,105 records identified, 7,065 were excluded following title and abstract screening. Forty studies underwent full-text evaluation and were double-checked by another assessor to avoid bias, of which nine met the inclusion criteria, encompassing a pooled cohort of 643 patients for final analysis. The PRISMA flow diagram is shown in Figure 1.

The primary clinical outcomes assessed included mortality, venous thromboembolism (VTE) recurrence, incidence of PE, and major bleeding events. Recurrent VTE was defined as recurrent UEDVT and DVT of the lower limbs or thrombosis occurring at unusual sites in most studies. Major bleeding was defined according to the criteria of the International Society of Thrombosis and Haemostasis in seven studies. PE was limited to symptomatic ones in most studies. We have used the Newcastle-Ottawa Scale to assess the quality of risk of bias in the included studies, which showed a 7/9 score given to historical LMWH data used and heterogeneous cohorts; however, this score indicates a low risk of bias.

## Review

Results

The nine studies included patients with UEDVT of varying etiologies [7-16], including idiopathic, coagulopathic, iatrogenic, central line-related, malignancy-associated DVT, and TOS-related DVT, as shown in Table 1.

**Table 1:** Studies included in the meta-analysis A, apixaban; Age-M, median of age; Ca ptn, number of patients with cancer on DOAC; Cath ptn, number of patients who had UEDVT secondary to central venous catheter; D, dabigatran; DOAC, direct oral anticoagulant; DOAC agent, number of patients on each DOAC drug; DOAC-N, number of patients given DOAC in each study; E, edoxaban; Male-N, number of male patients; R, rivaroxaban; Total-N, total number of patients in each study; UEDVT, upper extremity deep vein thrombosis; USA, United States of America

	Author	Study Design	Centres	Country	DOAC-N	Total-N	Age-M	Male-N	Ca ptn	Cath ptn	DOAC Agent
1	Davies et al. 2018 [8]	Prospective	Multicentre	Canada	70 (100.0%)	70	54	23 (32.9%)	70 (100.0%)	70 (100.0%)	R70
2	Fan et al. 2020 [9]	Prospective	Multicentre	China	44 (52.4%)	84	51	25 (29.8%)	84 (100.0%)	84 (100.0%)	R44
3	Fiori et al. 2020 [10]	Retrospective	Single centre	Italy	10 (100.0%)	10	28	3 (30.0%)	0 (0.0%)	0 (0.0%)	R5-A5
4	Houghton et al. 2017 [11]	Prospective	Single centre	USA	102 (48.6%)	210	60	124 (59.0%)	125 (59.5%)	107 (51.0%)	R39-A63
5	Laube et al. 2017 [12]	Retrospective	Single centre	USA	83 (100.0%)	83	62	32 (38.6%)	83 (100.0%)	83 (100.0%)	R83
6	Montiel et al. 2017 [13]	Retrospective	Multicentre	Sweden	55 (100.0%)	55	49	27 (49.1%)	10 (18.2%)	5 (9.1%)	R46-A7-D2
7	Porfidia et al. 2020 [14]	Retrospective	Multicentre	Italy	61 (100.0%)	61	49	32 (52.5%)	0 (0.0%)	0 (0.0%)	R37-A11-D7-E6
8	Schastlivtsev et al. 2019 [15]	Prospective	Single centre	Russia	30 (100.0%)	30	52	13 (43.3%)	2 (6.7%)	4 (13.3%)	R30
9	Vedovati et al. 2021 [16]	Prospective	Multicentre	Europe	188 (100.0%)	188	52	88 (46.8%)	55 (29.3%)	62 (33.0%)	R102-A56-D12-E18

Patients' characteristics

A total of 643 patients with UEDVT were treated with DOACs. The median age was 52 years, with 57.11% of the patients being male. Among the studied population, 66.72% had cancer-related UEDVT and 64.53% had catheter-related thrombosis, as shown in Table 2.

**Table 2:** Patients' characteristics DOAC, direct oral anticoagulant; UEDVT, upper extremity deep vein thrombosis

Characteristics	Value
Total patients with UEDVT treated with DOAC	643
Median age	52 years
Male percentage	57.11%
Percentage of patients with cancer	66.72%
Percentage of patients with a central line	64.53%
Median treatment period	3 months
Median follow-up period	6 months

Among the patients treated with DOACs, the distribution of specific agents was as follows: rivaroxaban (70.9%), apixaban (22%), edoxaban (3.7%), and dabigatran (3.3%). The median treatment duration was three months, while the median follow-up period extended to six months. The overall mortality rate among DOAC-treated patients was 2.49% (16/643). The recurrence rate of VTE was observed to be 0.93% (6/643), while the incidence of PE was relatively low at 0.31% (2/643). Additionally, the occurrence of major bleeding events was documented at 2.02% (13/643). These outcomes are summarized in Table 3.

**Table 3:** Primary outcomes calculated proportion of events and its 95% CI using the Clopper–Pearson exact method CI, confidence interval; VTE, venous thromboembolism

Outcome	Events (n)	Total Patients	Proportion (p)	95% CI
Death	16	643	2.49%	1.50%-3.98%
Recurrence of VTE	6	643	0.93%	0.38%-2.03%
Pulmonary embolism	2	643	0.31%	0.06%-1.12%
Major bleeding	13	643	2.02%	1.13%-3.42%

Statistical comparison with LMWH

The outcomes of DOAC-treated patients were compared with historical LMWH data for the four primary endpoints: mortality, VTE recurrence, PE, and major bleeding, as shown in Table 4.

**Table 4:** Comparison of DOAC vs. LMWH outcomes using the z-score DOAC, direct oral anticoagulant; LMWH, low-molecular-weight heparin; VTE, venous thromboembolism

Outcome	DOAC (%)	LMWH (%) (Median)	z-Score	p-Value	Significance
Mortality	2.49	22.0 (16.5-27.5)	-12.06	<0.001	DOAC significantly lower
VTE recurrence	0.93	5.0	-4.73	<0.001	DOAC significantly lower
Pulmonary embolism	0.31	6.5 (5-8)	-6.32	<0.001	DOAC significantly lower
Major bleeding	2.02	0.25 (2.5 per 1,000)	8.99	<0.001	DOAC significantly higher

Regarding mortality, the DOAC-treated cohort reported 16 deaths among 643 patients (2.49%). In contrast, LMWH-treated populations historically exhibited 30-day mortality rates, ranging from 16.5% to 27.5% in UEDVT patients [17]. A one-sample z-test confirmed this difference as statistically significant (z=−12.06, p<0.001), favoring DOACs.

Recurrent VTE occurred in six DOAC-treated patients (0.93%), whereas LMWH/warfarin regimens are associated with a 5% recurrence rate in UEDVT [17]. The z-test demonstrated statistical significance (z=−4.73, p<0.001). PE incidence was 0.31% (2/643) with DOACs compared to 5-8% in LMWH cohorts [17]. The difference was significant (z=−6.32, p<0.001). DOACs had a higher major bleeding rate (2.02%, 13/643) compared to LMWHs (0.25%, 2.5/1,000) [18]. This increase was statistically significant (z=8.99, p<0.001).

The Cochran’s Q, I² statistic, and p-values for heterogeneity across the nine studies in the meta-analysis for each outcome are as follows: 1. VTE recurrence: Q=6.10, I²=0%, p=0.64, so, there is no significant heterogeneity among studies for VTE recurrence. 2. PE: Q=2.02, I²=0%, p=0.98, it indicates extremely low variability; no heterogeneity detected. 3. Major bleeding: Q=13.37, I²=40.2%, p=0.10, a moderate heterogeneity is present. Although not statistically significant (p>0.05), variability across studies warrants caution in interpreting pooled results. The summary of data synthesis approach is shown in Table 5.

**Table 5:** Summary of data synthesis approach CI, confidence interval

Methodological Component	Description
Effect size	Proportions (e.g., recurrence rate, mortality)
CI method	Clopper–Pearson exact
Comparative test	One-sample z-test against historical controls
Heterogeneity	Calculated Q and I² separately
Software	Stata, version 12.0 (StataCorp LLC, Texas)
Limitations	Absence of data allowing random-effects model or subgroup analysis

Discussion

Interpretations

DOACs were associated with a significantly lower mortality rate (2.49% vs. 16.5-27.5%) [17]. Similarly, the recurrence rate of VTE was lower in the DOAC cohort (0.93%) compared to LMWH historical data (5%) [17]. The incidence of PE was also lower among DOAC-treated patients (0.31% vs. 5-8%) [17]. However, the major bleeding rate appeared to be higher with DOACs (2.02%) than with LMWH (0.25%) [18].

The findings suggest that DOACs may offer a favorable safety and efficacy profile in the management of UEDVT. Notably, the lower mortality and recurrence rates observed with DOACs, compared to historical LMWH data, highlight their potential advantage. However, the higher incidence of major bleeding associated with DOAC use raises concerns that warrant further investigation to explore bleeding risk modifiers (e.g., renal function, DOAC type).

Limitations

Several factors contribute to the challenges of studying UEDVT, including heterogeneity in patient populations, variations in the underlying etiology of thrombosis, and differences in anticoagulant regimens. The diverse pathophysiology of UEDVT, particularly catheter-associated and malignancy-associated cases, may influence treatment response and outcomes. Moreover, variations in follow-up protocols across studies make direct comparisons challenging.

The lack of routine imaging follow-up to assess venous patency limits the ability to determine the long-term impact of DOACs on clot resolution and post-thrombotic syndrome development. Future studies should aim to incorporate standardized imaging and longer follow-up periods to better evaluate the durability of treatment effects. We also acknowledge certain limitations of this study, including potential confounding variables of the patients' understanding predisposing factors, the use of various DOAC agents in the nine studies we included, and the reliance on historical data for LMWH comparisons. Small PE event numbers might limit statistical power to detect differences. Also, combining retrospective and prospective studies might bias pooled estimates.

## Conclusions

The available data suggest that DOACs may offer advantages over LMWHs in terms of lower mortality and PE incidence rates in UEDVT patients. However, because the available sources do not provide specific recurrence or major bleeding rates for LMWH across distinct DVT subgroups, such as cancer-related or catheter-related cases, we were unable to perform comprehensive statistical comparisons for these outcomes. As the advantages of DOACs in this study are based solely on indirect comparisons with historical controls, further randomized head-to-head trials with detailed outcome data are necessary to fully assess the comparative effectiveness and safety of DOACs versus LMWHs in UEDVT treatment.
